# Neuropeptides and Control of Food Intake

**DOI:** 10.1155/2014/910912

**Published:** 2014-04-02

**Authors:** Paolo de Girolamo, Carlos Dieguez

**Affiliations:** ^1^Department of Veterinary Medicine and Animal Productions, University of Naples Federico II, Via Veterinaria, 1, 80137 Naples, Italy; ^2^Universidade de Santiago de Compostela, Campus Vida, 15782 Santiago de Compostela, Spain

In all vertebrates, food intake is a sophisticated complex of neurohumoral networks that convey signals between the brain and periphery, to modulate energy status. Gut hormones, such as peptide YY, pancreatic polypeptide, glucagon-like peptide-1, oxyntomodulin, and ghrelin, are modulated by acute food ingestion. In contrast, adiposity signals such as leptin and insulin are implicated in both short- and long-term energy homeostases. The mechanisms of action of these substances are similar among vertebrates.

Their regulation might vary with the feeding and reproductive state, and between different tissues and organs, and it might also be affected by environmental parameters. The control of food intake is carried out by short-term and long-term regulation mechanisms. The short-term signals act primarily as determinants of satiety to limit the size of individual meals. Long-term signals communicate total energy stores, integrate over time, and interact with other systems that rely upon the energy status of the organism (e.g., growth, immune function, and reproduction). Both long- and short-term signals interact to influence the behavior and energy balance of the organism. We know that disrupted signaling in many of these systems leads to dramatic changes in feeding behavior and weight gain (or loss). However, fully understanding control of food intake will require knowledge of: (a) which peptides are involved; (b) areas of the central nervous system where this peptides are expressed and (c) assessment of the biological effects of the different neuropeptides in the integrated control of energy and metabolic homeostasis.

In this special issue we selected several papers that carry out a systematic and critical review of some of the topics that following recent developments are currently at the forefront of obesity research. This special issue is particularly timely since it becomes available 20 years after the seminal discovery of leptin in Friedman's lab.

H. M. Zerón et al. provide a deep and comprehensive review of the different gut hormones involved in energy homeostasis; they give an update on the available evidence regarding the interaction between macronutrients and gastrointestinal hormone secretion. Furthermore, they review the available data regarding the evidences postulating a yet uncharacterized production of a putative hormone produced in the foregut of diabetic patients that should act decreasing insulin-sensitivity at peripheral tissues. This topic is of particular clinical relevance since it provides a rationale for the so-called metabolic surgery that has been advocated by some as possible therapy in diabetic patients.

Most of the data gleaned over the last 20 years in energy homeostasis has been focused in the integrated control exerted at hypothalamic level between central and peripheral signals. The latter arises from the gastrointestinal tract, including the pancreas, and the adipose tissue. More recently it has become evident that proteins secreted for other peripheral tissues may also play an important role. Thus a new set of factors so-called myokines, produced by muscle and released into the blood circulation, has emerged as major regulators of energy and metabolic homeostasis. A recently discovered one, irsin, is postulated as a potential drug target due to its marked effects on energy expenditure and therefore body weight. The available data in this topic is reviewed by M. G. Novelle et al., who provide a critical assessment at the available evidences so far and some of the yet remaining questions regarding the mechanisms involved in its secretion and biological effects.

The intimate relationship between energy status and reproduction has been recognized for many years. Data gleaned more recently have clearly shown that signals, such as leptin and ghrelin, known to be involved in the regulation of energy homeostasis do also play an important role in the control of the hypothalamus-pituitary-gonadal axis. However, the mechanisms involved at central level mediating their effects have only recently become clearer. In this special issue, J. Roa provides an overview of the most recent developments in the field. In particular, a deep mechanistic insight assessing the implication of GnRH, Kiss1, NPY, and POMC neurons as the key factors contributing to the adaptation of the gonadal axis to different metabolic status is put forward.

Anorexia and bulimia nervosa are two clinical entities characterized by abnormal eating behavior. Despite many years of research, the mechanisms involved are far from being clarified. K. Smitka et al. extensively review the involvement of the different neuronal networks implicated in food intake and the available evidences regarding the alteration of different hormones and neuropeptides as potential physiopathological mechanisms in these two diseases. Furthermore they carry out a thorough assessment of the potential involvement of neutralizing autoantibodies against these peptides.

A topic that was neglected for many years but that is now at the forefront of research is the assessment of nutrition and anxiety. M. Murphy and J. G. Mercer review the most recent developments in the field. In particular, they critically review available data on the influence of different dietary components on anxiety-like behavior. Furthermore, they highlight the importance of fetal and neonatal programming and the limitations when comparing the outcomes of trials that involve differences in diet, species, strain, sex, and life stage, coupled with variation in duration, environment, and outcome measure.

Most of the studies regarding body weight homeostasis were focused in the issue of energy intake. Over the last few years considerable attention has been given to the study of mechanisms that promote or are stimulated by palatable food. J. R. Barson et al. addressed this issue by focusing on the two neuropeptides historically related to the lateral hypothalamus. They provide us with a deep insight into the mechanisms by which orexin and MCH promote the intake of palatable and/or caloric food and how the intake of these foods can influence the activity of the neurons producing these neuropeptides. In addition they describe their effects on the different brain area implicated in food reward.

